# The Prognostic Effect of CDKN2A/2B Gene Deletions in Pediatric Acute Lymphoblastic Leukemia (ALL): Independent Prognostic Significance in BFM-Based Protocols

**DOI:** 10.3390/diagnostics13091589

**Published:** 2023-04-28

**Authors:** Mirella Ampatzidou, Stefanos I. Papadhimitriou, Anna Paisiou, Georgios Paterakis, Marianna Tzanoudaki, Vassilios Papadakis, Lina Florentin, Sophia Polychronopoulou

**Affiliations:** 1 Department of Pediatric Hematology-Oncology (TAO), “Aghia Sophia” Children’s Hospital, 11527 Athens, Greece; 2 Laboratory of Hematology, Unit of Molecular Cytogenetics, “G. Gennimatas” General Hospital, 11527 Athens, Greece; 3 Bone Marrow Transplantation Unit, “Aghia Sophia” Children’s Hospital, 11527 Athens, Greece; 4 Laboratory of Flow Cytometry, Department of Immunology, “G. Gennimatas” General Hospital, 11527 Athens, Greece; 5 Department of Immunology, “Aghia Sophia” Children’s Hospital, 11527 Athens, Greece; 6 Alfa Laboratory Diagnostic Center, YGEIA Hospital, 11524 Athens, Greece

**Keywords:** acute lymphoblastic leukemia (ALL), child, genetics, CDKN2A/2B deletions, fluorescent in situ hybridization (FISH), multiple-ligation probe amplification (MLPA), copy-number alterations (CNAs), risk stratification, minimal residual disease (MRD)

## Abstract

One of the most frequent genes affected in pediatric ALL is the CDKN2A/2B gene, acting as a secondary cooperating event and playing an important role in cell-cycle regulation and chemosensitivity. Despite its inclusion in combined CNA (copy-number alterations) classifiers, like the IKZF1plus entity and the UKALL CNA profile, the prognostic impact of the individual gene deletions outside the context of a combined CNA evaluation remains controversial. Addressing the CDKN2A/2B deletions’ additive prognostic effect in current risk-stratification algorithms, we present a retrospective study of a Greek pediatric ALL cohort comprising 247 patients studied over a 24-year period (2000–2023). Herein, we provide insight regarding the correlation with disease features, MRD clearance, and independent prognostic significance for this ALL cohort treated with contemporary BFM-based treatment protocols. Within an extended follow-up time of 135 months, the presence of the CDKN2A/2B deletions (biallelic or monoallelic) was associated with inferior EFS rates (65.1% compared to 91.8% for the gene non-deleted subgroup, *p* < 0.001), with the relapse rate accounting for 22.2% and 5.9%, respectively (*p* < 0.001). The presence of the biallelic deletion was associated with the worst outcomes (EFS 57.2% vs. 89.6% in the case of any other status, monoallelic or non-deleted, *p* < 0.001). Survival differences were demonstrated for B-ALL cases (EFS 65.3% vs. 93.6% for the non-deleted B-ALL subgroup, *p* < 0.001), but the prognostic effect was not statistically significant within the T-ALL cohort (EFS 64.3 vs. 69.2, *p* = 0.947). The presence of the CDKN2A/2B deletions clearly correlated with inferior outcomes within all protocol-defined risk groups (standard risk (SR): EFS 66.7% vs. 100%, *p* < 0.001, intermediate risk (IR): EFS 77.1% vs. 97.9%, *p* < 0.001, high risk (HR): EFS 42.1% vs. 70.5% *p* < 0.001 for deleted vs non-deleted cases in each patient risk group); additionally, in this study, the presence of the deletion differentiated prognosis within both MRD-positive and -negative subgroups on days 15 and 33 of induction. In multivariate analysis, the presence of the CDKN2A/2B deletions was the most important prognostic factor for relapse and overall survival, yielding a hazard ratio of 5.2 (95% confidence interval: 2.59–10.41, *p* < 0.001) and 5.96 (95% confidence interval: 2.97–11.95, *p* < 0.001), respectively, designating the alteration’s independent prognostic significance in the context of modern risk stratification. The results of our study demonstrate that the presence of the CDKN2A/2B deletions can further stratify all existing risk groups, identifying patient subgroups with different outcomes. The above biallelic deletions could be incorporated into future risk-stratification algorithms, refining MRD-based stratification. In the era of targeted therapies, future prospective controlled clinical trials will further explore the possible use of cyclin-dependent kinase inhibitors (CDKIs) in CDKN2A/2B-affected ALL pediatric subgroups.

## 1. Introduction

Major survival improvements in pediatric acute lymphoblastic leukemia (ALL) have been accomplished through the refinement of the risk-adapted approach [1,2,3] and MRD-guided treatment [1,3,4], as well as due to the enhanced delineation of the underlying disease biology [5,6,7,8,9,10,11,12]. Apart from the well-established adverse genetic aberrations, like the BCR::ABL1 fusion and KMT2A gene rearrangements, modern therapeutic protocols are currently incorporating the combined evaluation of the copy-number status of selected genes, which may also serve as adverse modifiers [11,13,14]. Hence, although CNA classifiers like the IKZF1plus entity [13] and the UKALL CNA profile [14] are constantly gaining relevance as potential risk-stratification markers [15,16], the prognostic impact of individual single-gene deletions remains controversial in most cases.

One of the genes that has a disputable effect on prognosis in pediatric ALL is the cyclin-dependent kinase inhibitor 2A/2B (CDKN2A/2B), located on the 9p21 chromosomal region and comprising two tumor-suppressor genes lying adjacent to each other, which encode for three proteins: (a) p16^INK4A^ (inhibitor of CDK4), (b) p14^ARF^ (alternative reading frame) by CDKN2A, and (c) p15^INK4B^ by CDKN2B [17]. As a secondary cooperating event, inactivation of the CDKN2A/2B genes can play an important role in leukemogenesis, regulating the cell cycle, chemosensitivity, and apoptosis [17,18,19].

Although CDKN2A/B deletions are detected in approximately 20–25% of pediatric B-cell precursor (BCP) ALL cases and 38.5–50% of T-ALL patients [19,20,21], with the percentage rising to more than 80% in cases of B-other and BCR/ABL1-like ALL [22,23], results on the prognostic impact of the biallelic or monoallelic deletion remain inconclusive [24,25,26,27,28,29,30,31,32,33,34]. In addition, the use of cyclin-dependent kinase inhibitors (CDKIs) in CDKN2A/2B-affected ALL pediatric subgroups requires prospective evaluation in the framework of targeted therapies and controlled clinical trials. Herein, we present a retrospective study of a Greek pediatric ALL cohort studied over a 24-year period (2000–2023), with a median follow-up time of 135 months, providing insight regarding the deletion’s correlation with disease features and disease clearance and its independent prognostic significance in the context of contemporary BFM-based treatment protocols. Additionally, our study demonstrates that, in the absence of NGS technologies, the combination of iFISH and MLPA could be a simple, feasible, and validated approach for identifying the majority of CDKN2A/2B deletions.

## 2. Materials and Methods

### 2.1. Patients

During the years 2000–2023, 247 ALL patients (151 males/96 females, median age 5.0 years (range 0.2–17.5)) were consecutively diagnosed and homogeneously treated according to BFM-based protocols in a single center, the Department of Pediatric Hematology-Oncology (T.A.O.) of “Aghia Sophia” Children’s Hospital in Athens, Greece. The diagnosis of B-cell- or T-cell-precursor origin was established according to conventional FAB and immunophenotypic criteria. A total of 220 patients (89.1%) were diagnosed with B-cell-precursor ALL and 27 patients (10.9%) with T-cell-precursor ALL.

### 2.2. Diagnosis; Morphologic, Molecular, and Cytogenetic Testing

All patients were evaluated by morphology of bone-marrow (BM) smears, histochemistry, immunophenotyping, conventional cytogenetics (G-banding), FISH, and RT-PCR for the presence of common ALL translocations.

### 2.3. Flow Cytometry (FC)

BM samples were investigated for leukemia-associated immunophenotypes and were assessed by flow-cytometry (FC) using 3–5-color antibody combinations, adapted to the published AIEOP-BFM Consensus Guidelines 2016 for Flow Cytometric Immunophenotyping of Pediatric ALL for patients treated after 2016 [35]. Follow-up samples for minimal-residual-disease (MRD) study were collected from BM at days 15, 33, and 78; weeks 22–24 before initiation; and at the end of maintenance therapy. All high-risk (HR) patients were also evaluated before each HR block. MRD was detected by flow cytometry, initially using 5 colors and, since 2019, 9 and 10 colors for B-ALL and T-ALL phenotypes, respectively. Sample analysis was performed with FC-500 and NAVIOS (Beckman-Coulter, Miami, FL, USA) flow cytometers using CXP-Analysis or Kaluza (versions 1.3 and 2.1) software. For MRD detection, a minimum of 500,000 events was collected with count extrapolation of up to 3,600,000 events if needed. Sensitivity of 0.1 to 0.01% was achieved in most cases, with an acquisition of a minimum of 20 events in the MRD gate.

### 2.4. G-Banding, FISH, and RT-PCR

Bone-marrow cells were cultured for 24, 48, and 72 h prior to G-banding. A 300-banding resolution technique (300 bands per haploid set—300 bphs) was applied. FISH evaluation using commercial probe sets was performed in non-cultured cells for the detection of ETV6::RUNX1, TCF3::PBX1, and BCR::ABL1 fusion genes; KMT2A gene rearrangements; and CDKN2A/2B, ETV6, and RUNX1 duplications, deletions, or amplifications. Bone-marrow cells were analyzed with interphase FISH according to the probe manufacturer’ instructions (Abbott Molecular Inc., Abbott Park, IL, USA). The probe set employed consists of a centromeric probe for chromosome 9, plus a locus-specific identifier, measuring 222 kilobases (kb) and spanning the entire length of CDKN2A (INK4A and ARF) and CDKN2B (INK4B), as well as the entire length of the methylthioadenosine phosphorylase (MTAP) gene in the centromeric direction in the 9p21.3 chromosome region. Based on results from normal bone-marrow smears, the cutoff level for any kind of deletion or monosomy was set to 10%, and at least 300 cells were analyzed in each test. Cases with two different deleted populations (one biallelic and one monoallelic) were classified as having a biallelic deletion.

Ficoll-Hypaque-purified BM samples (Sigma-Aldrich, Saint-Louis, MO, USA, and Merck, Darmstadt, Germany) were studied by RT-PCR for the presence of the common translocations ETV6::RUNX1, TCF3::PBX1, BCR::ABL1, and KMT2A::AFF1.

### 2.5. MLPA (Multiple-Ligation Probe Amplification)

MLPA (multiple-ligation probe amplification) was applied using the SALSA-MLPA P335 kit (MRC Holland, Amsterdam, the Netherlands). Among the 247 ALL patients consecutively treated in our department (54 SR, 130 IR, 63 HR), BM samples from 95 non-selected patients were MLPA analyzed (retrospective: 45 patients, prospective and consecutively diagnosed since 2015: 50 patients), evaluating the copy-number status detection of 8 genes: IKZF1, CDKN2A/2B, PAR1, BTG1, EBF1, PAX5, ETV6, and RB1. The Salsa-MLPA-P335Kit was used according to the manufacturer’s instructions [36,37].

### 2.6. Conventional Risk Stratification, Therapy Groups, and Treatment Protocol

All patients were treated according to AIEOP-BFM-ALL-based protocols (BFM 1995/2000 and ALLIC-BFM 2009) [38,39,40]. Initial risk stratification was conducted according to protocol criteria [39,40]. All patients were stratified as good or poor prednisone responders (GPR or PPR) according to peripheral-blood (PB) smears on day 8 of remission-induction therapy (absolute-blast count < or ≥1000/µL).

Non-T ALL patients with WBC < 20,000/µL at diagnosis and age ≥ 1 to <6 years who lacked high-risk criteria and had an FC-MRD load on day 15 of <0.1% when treated on the ALLIC-BFM 2009 protocol were characterized as standard-risk (SR) patients according to protocol stratification. The high-risk (HR) group included patients with any of the following: detection of KMT2A/AFF1, detection of BCR/ABL1, poor prednisone response on day +8, inability to achieve complete remission (CR) on day +33, hypodiploidy, and FC-MRD ≥ 10% on day 15 for patients treated on the ALLIC-BFM 2009 protocol. All other patients were allocated to the intermediate-risk (IR) group by protocol stratification.

The remission induction, consolidation, and reinduction therapy was applied according to the BFM backbone, as previously described [41,42], using a two-arm BFM backbone applied before 2009 and following the three-arm ALLIC BFM 2009 stratification afterwards [41,42,43].

### 2.7. Statistical Analysis

Event-free survival (EFS) and overall-survival (OS) estimates were calculated using the Kaplan–Meier method and standard errors of the estimates were calculated using Greenwood’s formula. Time to relapse was calculated as the time from diagnosis to first relapse, whereas time to event was estimated as the time from diagnosis to the first adverse event (relapse, refractory disease, secondary malignancy, or death). Patients were censored at the time of last follow-up. OS was defined as the time from diagnosis to death from any cause, and patients were censored at the time of last follow-up. The log-rank test was used for comparison of survival curves between different groups. Multivariate analysis was conducted, and prognostic factors for EFS and OS were identified using the Cox proportional-hazard regression model. The significance of covariate or factor effects was tested using the Wald tests. Associations between categorical variables were tested using the x^2^ test. All tests were conducted with a significance level of 5% (*p*-values of ≤0.05 were considered statistically significant). Analysis was performed using IBM SPSS v29.0 software.

## 3. Results

### 3.1. FISH and MLPA Concordance in CDKN2A/2B Evaluation

In our cohort of 247 ALL patients, 63/247 patients (25.5%) harbored CDKN2A/2B deletions. The majority of CDKN2A/2B deletions were identified by FISH (55/63), with the rest of the cases detected by MLPA or karyotype. G-banding cytogenetics captured the deletion in only eight cases.

Among the 95 samples analyzed by MLPA in the whole cohort, 29 referred to the CDKN2A/2B-deleted subgroup, as identified by any method. Out of the 29 CDKN2A/2B-deleted samples evaluated, the deletion was identified in 23 cases by FISH and in 20 cases by MLPA. Concordance between FISH and MLPA was evidenced in 15 cases, eight cases were identified by FISH only, and five cases were detected by MLPA only, with negative FISH results.

### 3.2. The Incidence of CDKN2A/2B Deletions and Comparative Description of Clinical and Genetic Disease Features between the CDKN2A/2B Deleted and Non-Deleted Subgroup

Sixty-three out of 247 patients (25.5%) harbored CDKN2A/2B deletions, either biallelic (*n* = 35) or monoallelic (*n* = 28). Among 220 B-ALL patients, the presence of CDKNA/2B deletions was identified in 49/220 (22.3%), and within the 27 T-ALL subsets, CDKN2A/2B deletions were present in 14/27 patients (51.8%). The detection of CDKN2A/2B deletions was associated with older age at diagnosis (median age: 5.9 years vs. 4.3 years, *p* = 0.04), higher WBC count (median WBC: 22.15 × 10^9^/L vs. 9.33 × 10^9^/L, *p* < 0.001), and non-significant difference regarding CNS infiltration (12/63, 19.0% vs. 14.1% 26/184, *p* = 0.37) compared to the subgroup with non-deleted CDKN2A/2B. Regarding protocol risk stratification, patients harboring CDKN2A/2B deletions presented with a trend towards IR- and HR-group stratification, compared to patients without evidence of the aberration, with the SR group accounting for 14.3% within the deleted subgroup vs. 24.5% when analyzing the CDKN2A/2B-non-deleted subgroup. The presence of the deletion was associated with a higher co-occurrence of the BCR::ABL1 fusion transcript (4.8% vs. 1.1%) and the PAX5 gene deletion (13.8% vs. 6.1%). Comparative description of the deleted and non-deleted CDKN2A/2B subgroup and coexistence with other genetic aberrations is described in Table 1.

### 3.3. Impact of CDKN2A/2B Deletions in Treatment Response and MRD Clearance

A higher rate of poor prednisone response on day 8 of induction therapy was observed within the CDKN2A/2B-deleted subgroup (19.0% vs. 12.5% for non-deleted patients, *p* = 0.3), but the results were not statistically significant. No statistically significant differences were noted between the two genetic groups (CDKN2A/2B deleted vs. CDKN2A/2B non-deleted) regarding the prevalence of MRD positivity on days 15 and 33 (74.6% vs. 75.0% on day 15, *p* = 0.84 and 22.2% vs. 24.4% on day 33, *p* = 0.94). There was a trend for a higher percentage of end-induction complete remission (CR) in the CDKN2A/2B-deleted subgroup but with no statistically significant difference (*p* = 0.08).

The effect of the CDKN2A/2B deletions on early treatment response and MRD clearance is shown in Table 1.

### 3.4. Prognostic Impact of CDKN2A/2B Deletions on Survival Rates and Outcome

With a median follow-up time of 135 months, overall survival (OS) and event-free survival (EFS) for the whole cohort were 89.9% and 85.0%, respectively. EFS rates for B-ALL and T-ALL patients were 87.3% and 66.7%, respectively (*p* = 0.002).

The presence of the CDKN2A/2B deletion (biallelic or monoallelic) was associated with inferior EFS of 65.1% compared to 91.8% for the gene-non-deleted subgroup (*p* < 0.001), with a relapse rate of 22.2% and 5.9% for the deleted and non-deleted cases, respectively (*p* < 0.001).

Patients that harbored a biallelic deletion had EFS rates of 57.2% vs. 89.6% in the case of any other status (monoallelic or non-deleted) (*p* < 0.001). In the case of patients in whom the deletion was monoallelic, EFS was 73.1% compared to 86.4% for the rest of the cohort (*p* = 0.124). Focusing solely on the CDKN2A/2B-deleted subgroup and further analyzing the gene-allelic status within the deleted sub-cohort, biallelic deletion was associated with adverse outcomes compared to the monoallelic aberration (EFS of 57.1% vs. 75.0%, *p* = 0.002).

Among the B-ALL cohort, the presence of the CDKN2A/2B deletion was associated with inferior outcomes (EFS 65.3% vs. 93.6% for the non-deleted B-ALL subgroup, *p* < 0.001) and a relapse rate of 24.5% vs. 5.8%, respectively (*p* < 0.001).

Analyzing the T-ALL cohort separately, CDKN2A/2B-deleted patients had non-statistically significant survival differences compared to their T-ALL non-deleted counterparts (EFS 64.3 vs. 69.2, *p* = 0.947).

**Figure 1:**
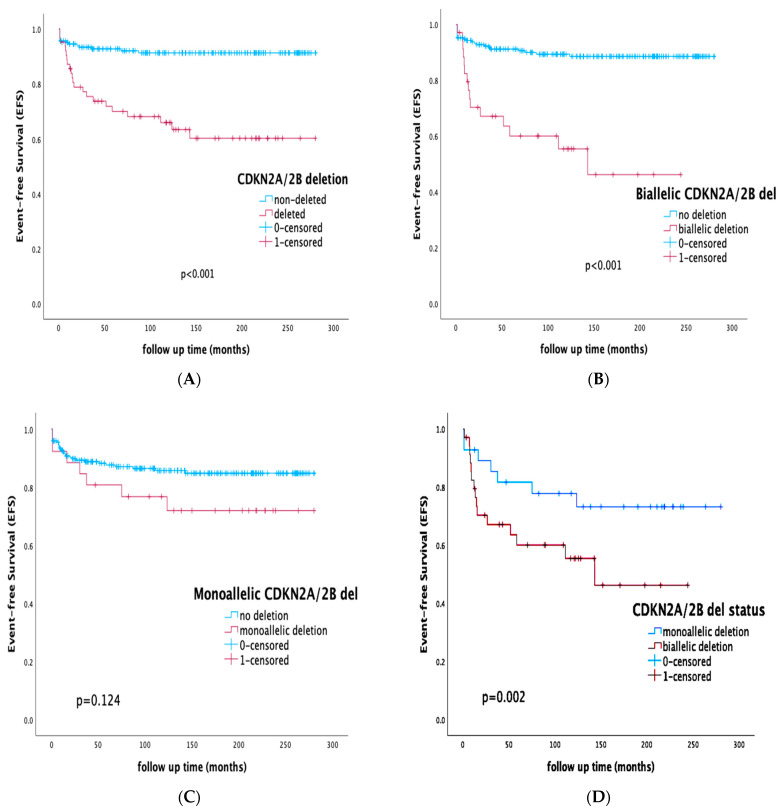

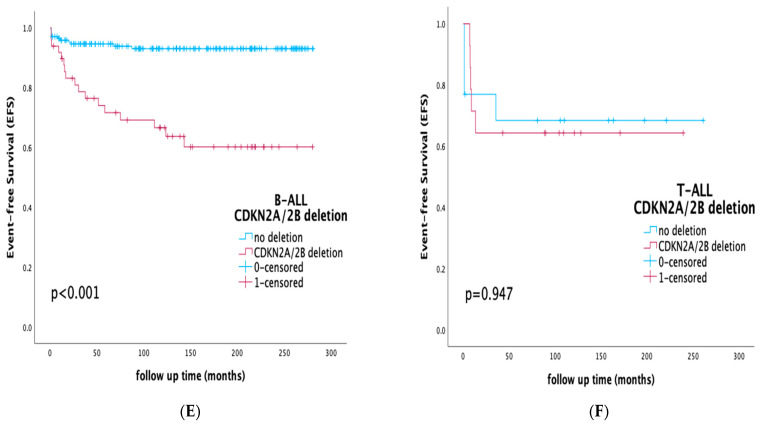
Event-free survival (EFS) rates in specific cohorts: (**A**) whole cohort, EFS of the CDKN2A/2B-deleted vs. -non-deleted subgroup; (**B**) whole cohort, EFS of the CDKN2A/2B-biallelic-deleted vs. -non-deleted subgroup; (**C**) whole cohort, EFS of the CDKN2A/2B-monoallelic-deleted vs. -non-deleted subgroup; (**D**) CDKN2A/2B-deleted subgroup, EFS by the status of CDKN2A/2B deletion, biallelic vs. monoallelic; (**E**) B-ALL cohort, EFS of the CDKN2A/2B-deleted vs. -non-deleted subgroup; (**F**) T-ALL cohort, EFS of the CDKN2A/2B-deleted vs. -non-deleted subgroup.

Survival rates of specific cohorts by the presence of CDKN2A/2B deletion are presented in Figure 1.

### 3.5. Prognostic Impact of CDKN2A/2B Deletions by Risk Stratification and Integration of MRD Status

**Figure 2:**
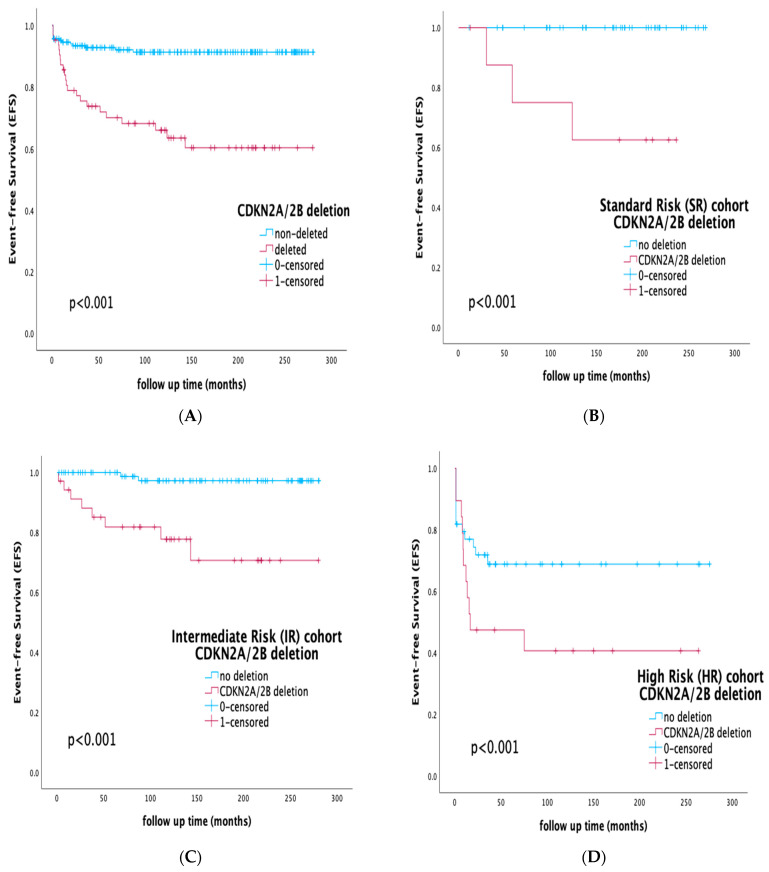
Event-free survival (EFS) rates by CDKN2A/2B deletion within separate therapy risk groups: (**A**) whole cohort, EFS of the CDKN2A/2B-deleted vs. -non-deleted subgroup, (**B**) standard-risk-group (SR) cohort, EFS of the CDKN2A/2B-deleted vs. -non-deleted subgroup, (**C**) intermediate-risk-group (IR) cohort, EFS of the CDKN2A/2B-deleted vs. -non-deleted subgroup, (**D**) high-risk-group (HR) cohort, EFS of the CDKN2A/2B-deleted vs. -non-deleted subgroup.

The presence of the CDKN2A/2B deletion also further stratified patients within all conventional risk groups, as defined by the BFM-protocol stratification. Within the SR group, the presence of the deletion was associated with inferior outcomes of only 66.7% vs. 100% for the rest of the SR patients (*p* < 0.001). Similarly, within the IR and HR groups, EFS for the CDKN2A/2B-deleted subgroup was 77.1% and 42.1%, respectively, compared to 97.9% and 70.5%, respectively, for IR and HR patients who did not harbor the deletion (*p* < 0.001). Survival rates by CDKN2A/2B deletion within separate therapy risk groups are shown in Figure 2.

**Figure 3:**
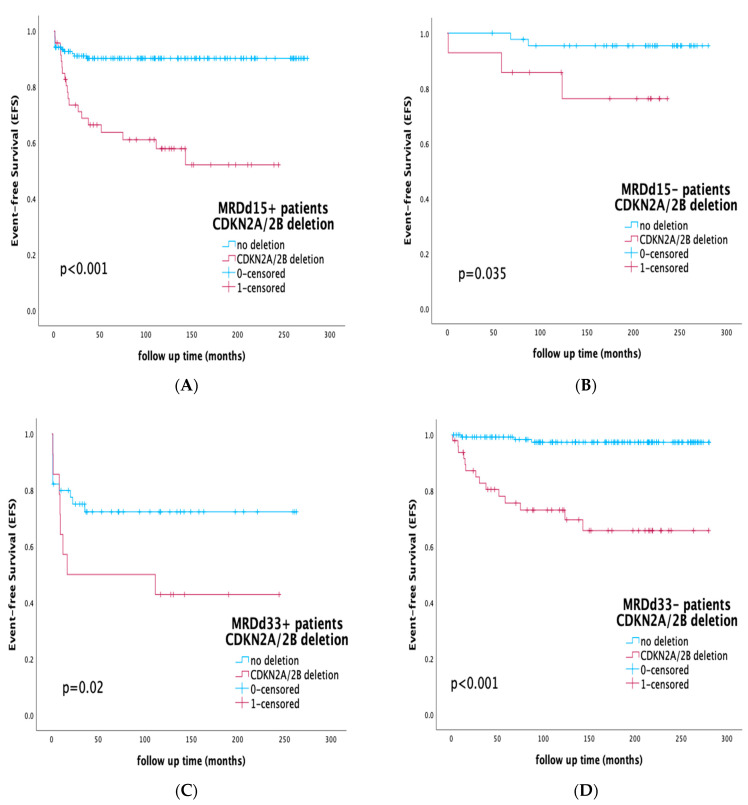
Event-free survival (EFS) rates by CDKN2A/2B deletion within separate MRD subgroups: (**A**) MRDd15+ patients on day 15 of induction, EFS of the CDKN2A/2B-deleted vs. -non-deleted subgroup; (**B**) MRDd15− patients on day 15 of induction, EFS of the CDKN2A/2B-deleted vs. -non-deleted subgroup; (**C**) MRDd33+ patients on day 33 of induction, EFS of the CDKN2A/2B-deleted vs. -non-deleted subgroup; (**D**) MRDd33− patients on day 33 of induction, EFS of the CDKN2A/2B-deleted vs. -non-deleted subgroup.

To evaluate the prognostic effect of the CDKN2A/2B deletion within distinct MRD subgroups, we analyzed the presence of the deletion within MRD-positive and -negative subgroups on days 15 and 33 of induction therapy. The detection of CDKN2A/2B deletion further stratified patients both within the MRDd15-positive subgroup on day 15 (EFS 59.6% vs. 90.6%, *p* < 0.001) and within the MRDd15-negative subgroup (EFS 78.6% vs. 95.6%, *p* = 0.035). Additionally, analyzing the cohort by MRD status at the end of induction (day 33), the presence of CDKN2A/2B deletion further stratified both the MRDd33-positive and MRDd33-negative subgroups, respectively. Within the MRDd33-positive subgroup, CDKN2A/2B-deleted cases had inferior outcomes (EFS 42.9% vs. 72.3% for the non-deleted MRDd33-positive cases, *p* = 0.02), and similar statistically significant differences were found within the MRDd33-negative sub-cohort (EFS 71.5% vs. 97.8%, *p* < 0.001). Survival rates by CDKN2A/2B deletion within separate MRD subgroups are displayed in Figure 3.

### 3.6. Mutivariate Analysis and Correlation with Protocol Conventional Risk Factors

In an attempt to define the interaction between the presence of the CDKN2A/2B deletion, MRD, and other conventional risk factors, multivariate analysis was conducted and Cox regression analysis for EFS and OS was performed with the following covariables: presence of CDKN2A/2B deletions, FC-MRD status on day 15, FC-MRD status on day 33, BCR/ABL1 status, KMT2A status, and protocol-risk-group stratification.

The presence of the CDKN2A/2B deletions was the most important prognostic factor for relapse, yielding a hazard ratio of 5.2 (95% confidence interval: 2.59–10.41, *p* < 0.001). Treatment-risk-group allocation and positive FC-MRDd33 status at the end of induction were also prognostic for relapse, with a hazard ratio of 3.85 (95% confidence interval: 1.58–9.35, *p* = 0.003) and 2.5 (95% confidence interval: 1.13–5.53, *p* = 0.024), respectively. 

Regarding OS, the presence of the CDKN2A/2B deletion was the most important prognostic factor for survival, yielding a hazard ratio of 5.96 (95% confidence interval: 2.97–11.95, *p* < 0.001), with risk-group allocation also retaining prognostic significance for survival, with a hazard ratio of 5.66 (95% confidence interval: 2.18–14.64, *p* < 0.001).

Details regarding multivariate Cox-regression analysis are shown in Table 2.

## 4. Discussion

During the past decade, the evolvement of genome-wide technologies and the identification of gene copy-number alterations (CNAs) implicated in leukemogenesis have led to a constant decoding of the underlying biology of pediatric ALL [5,6,8,12]. One of the most frequent genes affected is the CDKN2A/2B gene, acting as a secondary cooperating event and playing an important role in cell-cycle regulation and chemosensitivity [8,11,18].

In the current study, we addressed CDKN2A/2B deletions’ disputable prognostic significance [17,24,25,26,27,28,29,30,31,32,33,34] and provided evidence on its additive prognostic effect in current risk-stratification algorithms. We showed that the presence of the deletion is an independent prognostic factor and can further stratify all existing risk groups, integrating with MRD and identifying patient subgroups with different outcomes.

The CDKN2A/2B deletions are the most frequent CNAs in pediatric ALL, with most published studies reporting incidence rates of 20–25% in B-cell-precursor (BCP) ALL and 38.5–50% in T-ALL cases [17,18,19,21]. In our study, the CDKN2A/2B deletion accounted for 25.5% of the whole cohort (22.3% among B-ALL cases), with the prevalence of the deletion rising to 51.8% when evaluated within the T-ALL subgroup. As expected, the percentage detected directly correlates with the genomic-methodology technique applied, since the deletion can be detected by conventional cytogenetics, iFISH, MLPA, array-based comparative genomic hybridization (aCGH), and single-nucleotide polymorphism arrays (SNP-arrays) [19,20,44,45,46]. Some homozygous deletions in the 9p21 region might be the result of a heterozygous deletion followed by a copy-neutral loss of heterozygosity (CN-LOH), often referred to as uniparental disomy (UPD) [18,20,30]. This is an underappreciated chromosomal defect by conventional cytogenetics tools [18]. In our study, apart from cytogenetics, combined iFISH and MLPA evaluation was used, with iFISH identifying the CNAs in 87.3% of the positive cases; concordance between the two methods was 51.7%. The discordance in the identified results could be attributed to differences in cut-off sensitivity, presence of the deletion in minor subclones, or very small deletions that could be missed due to the size of probes used. The major limitations concern the MLPA method, which may not be sensitive enough for the detection of low-level (<20%) or mixed-cell populations, for which FISH is a more reliable technique [18,36,37,46]. Nevertheless, despite the fact that novel technologies such as aCGH and SNP-arrays could possibly overcome technique limitations [20,44], our study suggests that the combination of iFISH and MLPA could be a simple, feasible, and validated approach for identifying the majority of deletions.

In concordance with previously published reports [17,18,19], the presence of CDKN2A/2B deletions was associated with older age (median: 5.9 vs. 4.3 years, *p* = 0.04), higher WBC count upon diagnosis (median WBC: 22.15 × 10^9^/L vs. 9.33 × 10^9^/L, *p* < 0.001), and a trend towards IR- and HR-group stratification. The presence of the deletion was also associated with a higher co-occurrence of the BCR/ABL1 fusion transcript (4.8% vs. 1.1%, *p* = 0.04) and PAX5 gene deletion (13.8% vs. 6.1%, *p* = 0.04). The extent to which the presence of the abovementioned disease features translates to inferior outcomes of the CDKN2A/2B-deleted subgroup was one of the main scopes of our study.

CDKN2A/2B deletion has recently been incorporated into combined can-risk algorithms and classifiers, like the IKZF1plus entity [13] and the UKALL CNA profile [14]. The evaluation of CDKN2A/2B-deletion status, allocating patients to the CNA-poor-risk (CNA-PR) genomic subgroup, was part of a combined CNA algorithm introduced by Moorman et al. in the UKALL trials [14]. In the previously published study of our group [15], we demonstrated that the implementation of this can-profile risk index, including CDKN2A/2B gene status, could be feasible in BFM-based protocols, effectively stratifying patients within all conventional risk subgroups and identifying subsets of different prognosis. Despite its inclusion in combined algorithms, depending on the presence or absence of concurrent deletions, the prognostic impact of individual gene deletions outside the context of combined CNA evaluation remains controversial [17,18,19,20,21]. Many researchers have supported that CDKN2A/B deletions in childhood ALL were associated with an increased probability of relapse and impaired outcome [17,18,19,21,24,25,26,27,28,29,30,31], whereas Mirebeau et al. [32], Kim et al. [33], and van Zutven et al. [34] concluded that the presence of the deletion was not a poor prognostic factor in childhood B-ALL. In our study, within an extended follow-up time of 135 months, the presence of the CDKN2A/2B deletion (biallelic or monoallelic) was associated with inferior EFS rates (65.1% compared to 91.8% for the gene-non-deleted subgroup, *p* < 0.001), with the relapse rate accounting for 22.2% and 5.9% of the deleted and non-deleted cases, respectively (*p* < 0.001). Although the presence of the deletion was associated with a higher CR rate by the end of induction, this was not statistically significant, and the impact on EFS comes from the higher incidence of relapses, possibly due to acquired chemoresistance and clonal evolution. When addressing the specific prognostic value based on allelic status, many studies have supported that any loss of CDKN2A/2B tumor-suppressor genes may serve as an adverse prognostic marker [17,19,21,26,29], with others disputing the independent prognostic significance in the case of heterozygosity and coexisting aberrations [25,30,32,33,34]. Our results demonstrate that the presence of the biallelic deletion was associated with worst outcomes, (EFS 57.2% vs. 89.6% in the case of any other status, monoallelic or non-deleted, *p* < 0.001), and direct comparison between biallelic and monoallelic status revealed statistically significant differences in outcome and relapse prediction (EFS 57.1% vs. 75.0%, respectively *p* = 0.002).

Another interesting finding of our study was the fact that the presence of CDKN2A/2B deletions served as an important prognostic marker in B-ALL (EFS 65.3% vs. 93.6% for the non-deleted B-ALL subgroup, *p* < 0.001), but the prognostic effect was not statistically significant within the T-ALL cohort (EFS 64.3 vs. 69.2, *p* = 0.947). Although many studies have supported the independent prognostic significance of CDKN2A/2B deletions in adult T-ALL [47,48], the spectrum of genomic heterogeneity in pediatric T-ALL has still not been fully explored [49,50,51]. It is possible that the adverse prognosis in T-ALL is mainly driven by a variety of initiating and cooperating events, coinciding with heterogenous underlying mechanisms. These mechanisms may include CDKN2A/2B gene-promoter hypermethylation leading to downregulation, the absence of a biallelic deletion (ABD), variable co-deletion of contiguous genes like the methylthioadenosine phosphorylase (MTAP) cluster that are not always identified, and impaired myocyte enhancer factor 2C (MEF2C) expression, all associated with various impacts on chemosensitivity and drug resistance [52,53,54].

The major challenge in our study was to demonstrate CDKN2A/2B deletions’ prognostic significance within all already-established risk groups. It is noteworthy that the majority of ALL recurrences were still observed in the large group of IR patients. In the AIEOP-BFM ALL 2000 protocol, 69% of relapses occurred in IR patients [55], highlighting the need for additional prognostic markers in this heterogenous, not-well-defined spectrum of IR subsets. In our studied cohort, the presence of the deletion was not associated with statistically significant differences in terms of MRD clearance and CR achievement (Table 1), but it clearly correlated with inferior outcomes within all protocol-defined risk groups (EFS SR: 66.7% vs. 100%, *p* < 0.001, IR: 77.1% vs. 97.9%, *p* < 0.001, HR: 42.1% vs. 70.5% *p* < 0.001). The results of our study demonstrate that the evaluation of CDKN2A/2B deletions can identify a subgroup of adverse-prognosis patients within the SR and IR treatment groups who may benefit from early treatment intensification. In the context of MRD-guided treatment protocols and integrating with MRD, the presence of the deletion in our patient cohort also effectively stratified MRD-positive and -negative subgroups on days 15 and 33 of induction therapy (Figure 3), suggesting that even among MRD-negative patients, by the end of induction the presence of the deletion served as an adverse modifier, moderating prognosis and outcome.

In multivariate analysis, the presence of CDKN2A/2B deletions was the most important prognostic factor for relapse and overall survival, yielding a hazard ratio of 5.2 (95% confidence interval: 2.59–10.41, *p* < 0.001) and 5.96 (95% confidence interval: 2.97–11.95, *p* < 0.001), respectively, designating the deletion’s independent prognostic significance in the context of modern risk stratification.

## 5. Conclusions

In the current study, the presence of CDKN2A/2B deletion, especially in the case of biallelic status, was associated with inferior outcomes in B-ALL, with subcohorts of different prognosis identified within all conventional risk groups. In multivariate analysis, the presence of CDKN2A/2B deletion retained independent prognostic significance, representing a novel proposed factor for predicting relapse and survival. The identification of the deletion is low cost, simple, and feasible via a combination of iFISH and MLPA, leading to early identification of distinct patient subgroups with different prognosis. The results indicate that biallelic CDKN2A/2B deletion can be a genomic feature incorporated into future risk-stratification algorithms in an effort to further genetically refine MRD-based stratification and improve treatment-group allocation and ultimate patient outcome. In the frame of targeted therapies, future prospective controlled clinical trials should explore the use of cyclin-dependent kinase inhibitors (CDKIs) [56,57,58] in CDKN2A/2B-affected ALL pediatric subgroups.

## Figures and Tables

**Table 1:** Comparison of baseline demographic, clinical, immunophenotypic, genetic, and treatment characteristics of ALL patients with or without the presence of CDKN2A/2B deletions.

	Total (*N* = 247)	Patients with CDKN2A/2B Deletions (*N* = 63)	Patients without CDKN2A/2B Deletions (*N* = 184)	*p*-Value
**Characteristics**	*n* (%)	*n* (%)	*n* (%)	
**Gender**				
Male	151 (61.1)	40 (63.5)	110 (59.8)	
Female	96 (38.9)	23 (36.5)	74 (40.2)	0.92
**Age**				
Median, years	5.0	5.9	4.3	0.04
**Immunophenotype**				
B-ALL	220 (89.1)	49 (77.8)	171 (92.9)	
T-ALL	27 (10.9)	14 (22.2)	13 (7.1)	<0.001
**White-Blood-Cell Count**				
Median (×10^9^/L)	12.21	22.15	9.33	<0.001
**CNS Infiltration**				
Yes (CN2, CN3)	38 (15.4)	12 (19.0)	26 (14.1)	0.37
**Genetics**				
ETV6::RUNX1	50 (20.2)	10 (15.9)	40 (21.7)	0.32
KMT2A rearrangements	12 (4.8)	1 (1.6)	11 (6.0)	0.03
BCR::ABL1	5 (2.0)	3 (4.8)	2 (1.1)	0.04
TCF3::PBX1	10 (4.0)	3 (4.8)	7 (3.8)	0.97
iAMP21	3 (0.8)	1 (1.6)	2 (1.1)	0.78
Hyperdiploidy	61 (24.7)	10 (15.9)	51 (27.7)	0.06
Hypodiploidy	2 (0.8)	1 (1.6)	1 (1.1)	0.90
IKZF1deletion	13 (13.7) *	2 (6.9) **	11 (16.7) ***	0.02
IKZF1plus	1 (1.0) *	0 (0.0)	1 (1.5) ***	0.31
PAX5 deletion	8 (8.4) *	4 (13.8) **	4 (6.1) ***	0.04
**Treatment Protocol**				
BFM 95/2000 modified	119 (48.2)	31 (49.2)	88 (47.8)	0.95
ALLIC BFM 2009	128 (51.8)	32 (50.8)	96 (52.2)	0.65
**Protocol Risk Group**				
Standard risk	54 (21.9)	9 (14.3)	45 (24.5)	0.03
Intermediate risk	130 (52.6)	35 (55.6)	95 (51.6)	0.08
High risk	63 (25.5)	19 (30.1)	44 (23.9)	0.09
**Therapy Risk Group**				
Standard risk	7 (2.8)	0 (0)	7 (3.8)	0.04
Intermediate risk	167 (67.6)	43 (68.3)	124 (67.4)	0.09
High risk	73 (29.6)	20 (31.7)	53 (28.8)	0.06
FC-MRD status				
FC-MRDd15 positive (MRD_d15_ > 10^−4^)	185 (74.9)	47 (74.6)	138 (75.0)	0.84
FC-MRDd33 positive (MRD_d33_ > 10^−4^)	59 (23.9)	14 (22.2)	45 (24.4)	0.94
**Complete Remission (EOI-CR #)**				
Yes	230 (93.1)	61 (96.8)	167 (90.8)	0.08
No	17 (6.9)	2 (3.2)	17 (9.2)	0.06

* Results out of 95 BM samples evaluated by MLPA; ** results out of 29 BM samples evaluated by MLPA; *** results out of 66 BM samples evaluated by MLPA; FC-MRD: flow cytometry–minimal residual disease; EOI-CR: end of induction–complete remission; # complete remission, defined as flow-cytometric evaluation of <1% lymphoblasts by the end of induction.

**Table 2:** (**A**) EFS multivariate Cox-regression analysis; (**B**) OS multivariate Cox-regression analysis, with inclusion of the covariables listed in the table.

(**A**)						
	**SE**	**Wald**	**Sig.** ***p*-Value**	**Hazard Ratio (HR)**	**95.0% CI for HR**	
					**Lower**	**Upper**
CDKN2A/2B deletion	0.354	21.641	<0.001	5.199	2.596	10.412
BCR/ABL1+	0.656	0.003	0.958	0.966	0.267	3.493
KMT2A+	0.637	0.140	0.708	1.269	0.364	4.421
Τ vs. B ALL	0.416	0.000	1.000	1.000	0.443	2.258
MRDd15 positivity	0.564	0.046	0.830	0.886	0.293	2.678
MRDd33 positivity	0.405	5.118	0.024	2.501	1.130	5.535
Therapy risk group	0.453	8.867	0.003	3.851	1.585	9.353
(**B**)						
	**SE**	**Wald**	**Sig.** ***p*-Value**	**Hazard Ratio (HR)**	**95.0% CI for HR**	
					**Lower**	**Upper**
CDKN2A/2B deletion	0.355	25.260	<0.001	5.958	2.970	11.949
BCR/ABL1+	0.688	0.161	0.688	0.759	0.197	2.920
KMT2A+	0.645	0.064	0.800	1.177	0.332	4.170
Τ vs. B ALL	0.430	0.054	0.817	0.905	0.389	2.104
MRDd15 positivity	0.570	0.001	0.979	1.015	0.332	3.102
MRDd33 positivity	0.407	3.355	0.067	2.106	0.949	4.674
Therapy risk group	0.485	12.755	<0.001	5.657	2.186	14.640

## Data Availability

The authors ensure that the data shared are in accordance with consent provided by participants on the use of confidential data. The data presented in this study are available on request from the corresponding author. Hard copies of all data and results are also available in the patients’ files and collaborating involved laboratories. The data are not publicly available due to privacy and ethical restrictions.
